# VisuMax Flap 2.0: a flap plus technique to reduce incidence of an opaque bubble layer in femtosecond laser–assisted LASIK

**DOI:** 10.1007/s00417-022-05894-1

**Published:** 2022-11-14

**Authors:** Zichen Wang, Xinliang Cheng, Xueying Lou, Hongliang Chen, Zhifeng Lu, Hui Chen, Ying Yu

**Affiliations:** Department of Ophthalmology, Affiliated Hospital of Nantong University, Medical School of Nantong University, Nantong, 226001 China

**Keywords:** Opaque bubble layer, LASIK, Femtosecond laser, Flap

## Abstract

**Purpose:**

To evaluate the incidence of an opaque bubble layer (OBL) in femtosecond laser–assisted in situ keratomileusis (FS-LASIK) flaps created with VisuMax Flap 2.0 as a result of a modification in the parameters of the flap programming.

**Methods:**

This retrospective study was comprised of 1400 eyes of 715 patients who received FS-LASIK surgery. OBLs were measured and reported as a percentage of the flap area to identify the incidence and extent. Flap creation, which is a modification technique, was performed with 8.1-mm flap diameters plus 0.3-mm enlarged interlamellar photodisruption (group Flap 2.0). The same flap diameters without extra photodisruption as the previous standard setting were also implemented (group Flap 1.0). The preoperative measurements, including sphere, cylinder, keratometry, and intraoperative characteristics such as flap size and thickness, were documented. Possible risk factors for the occurrence of OBLs were investigated in this study.

**Results:**

The incidence of an OBL was reduced when using the Flap 2.0 program (31.4%) compared to the Flap 1.0 program (63.7%). The area of hard and soft OBLs created by the Flap 2.0 program is smaller than those created by the Flap 1.0 program (*P* = 0.007 and *P* < 0.001). Multivariate logistic regression indicated that a thinner flap (*P* = 0.038) and a higher sphere (*P* = 0.001) affected the chance of hard OBLs occurring.

**Conclusion:**

The VisuMax Flap 2.0 program promotes gas venting by enlarging the interlamellar photodisruption size. The incidence and extent of OBLs appear to be reduced significantly when the Flap 2.0 program is applied.


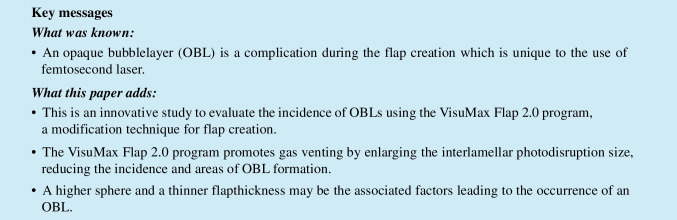


## Introduction

Femtosecond laser–assisted in situ keratomileusis (FS-LASIK) is widely used in contemporary corneal refractive surgery as an effective means of correcting myopia and myopic astigmatism. The creation of the corneal flap is a key step in the surgery procedure. Femtosecond laser flap creation allows a surgeon to achieve greater choice of intraoperative parameters, flap thickness, flap diameter, side-cut angle, and hinge position may all be customized, making this surgery safer, more predictable, and more accurate over conventional microkeratome-assisted procedures [1, 2]. The femtosecond laser creates microplasma in the corneal stroma by generating ultrashort pulses, forming an intrastromal cleavage plane to separate the corneal stroma by cavitation bubbles [3]. After these cavitation bubbles collapse, most of them disappear during the flap lifting process. However, some gas bubbles, which consist of carbon dioxide and water vapor, can be left behind. An opaque bubble layer (OBL) is generated by gas bubbles that cannot be expelled and accumulate in the superficial layers of the corneal stroma bed, resulting in widespread tissue opacity [1, 4–6].

OBLs are one of the most common complications from intraoperative femtosecond laser–assisted LASIK [7, 8]. Epithelial gas breakthrough is a rare but serious complication of OBLs [9]. The microbubbles may break vertically through the corneal epithelium during flap creation if the femtosecond laser cannot photodisrupt corneal stroma in a small portion of the intended interface or if there is resistance within the interface from scar tissue, leading to corneal tearing, incomplete flaps, buttonholing, and even the failure of the surgery procedure [7, 10].

According to the characteristics and the time of occurrence of an OBL, they are divided into two types: hard OBLs, which appear early and have a denser structure, and soft OBLs, which appear later and have a more transparent shape [3, 11]. The presence of an OBL may present challenges for flap separation and lifting. The pupil picture utilized by excimer laser trackers is temporarily obscured by an OBL and the patient’s fixation target during laser ablation, subsequently interfering with the surgical procedure and the precision of the femtosecond laser tissue separation [12, 13]. OBL is hard to be removed by sweeping the stroma with a Weck cell sponge after the flap lift [11]. During the surgery, using a soft docking technique with a relatively wide diameter flap helps to dock the patient interface just enough to leave a peripheral ring of tear meniscus, allowing gas bubbles to dissipate through the side cut [7, 11]. If the method does not work and there is still an OBL, excimer therapy can be postponed to give the bubbles time to escape [11].

In order to analyze the occurrence of OBLs, many corneal characteristics and surgical parameters were investigated, including different flap diameters, flap shapes, hinge angles, and sizes of cone [1, 12, 14]. The purpose of this study was to assess the incidence of OBL and the parameters that influence flap creation designed with VisuMax Flap 2.0, which changes the parameters of interlamellar photodisruption diameters in flap programming.

## Methods

This was a retrospective comparative study. The eyes of consecutive patients who underwent LASIK eye surgery were evaluated with corneal flaps created by a femtosecond laser for myopia or myopic astigmatism at Affiliated Hospital of Nantong University from July 2020 and July 2021. The study was performed according to the tenets of the Declaration of Helsinki and approved by Ethics Committee of Affiliated Hospital of Nantong University. The inclusion criteria were as follows: a minimum age of 18 years, a positive spherical refractive error of less than 10.50 diopter (D) with or without a cylindrical refractive error of less than 6D, a corrected distance visual acuity (CDVA) better than 0.10 logarithm of the minimum angle of resolution (log MAR). Patients who wore soft or rigid contact lenses were told to take them off for at least 2 and 4 weeks before the FS-LASIK surgery, respectively. Patients with a history of ocular surgery or trauma, keratoconus, other eye inflammatory diseases such as uveitis or sclerositis, systemic diseases such as diabetes, hypertension, cardiovascular diseases, or autoimmune diseases, pregnancy or lactation, or residual stromal thickness of less than 305 μm at the thinnest point were all considered exclusion criteria. The surgical log was used to gather all of the eyes that were videotaped while having LASIK flap formation with the VisuMax laser by a single surgeon (YY). According to the different flap creation techniques, all eyes were divided into two groups: group Flap 1.0 and group Flap 2.0. In each group, eyes were divided into a “no OBL” group, a “hard OBL” group, and a “soft OBL” group according to the presence or absence and the types of OBL.

### Surgical technique

**Fig. 1:**
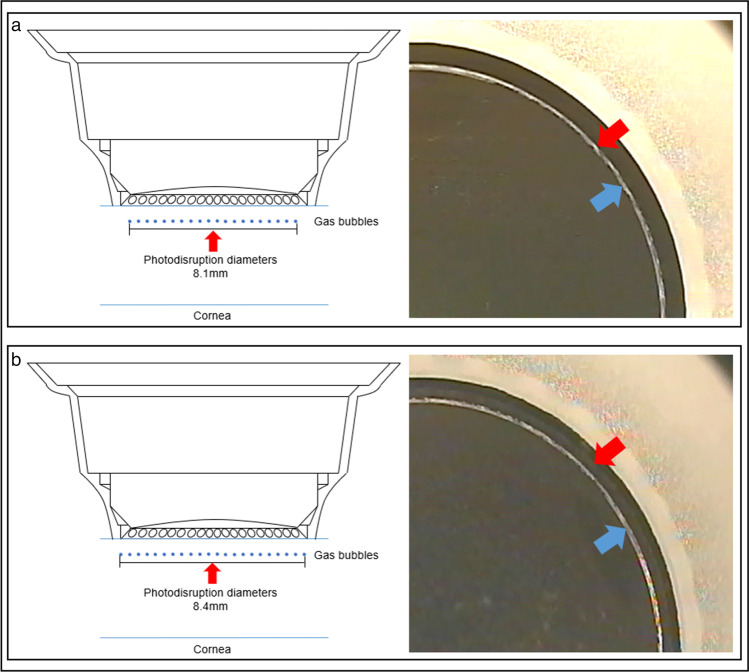
Image showing two representative flaps. **a** Flap creation in group Flap 1.0: the photodisruption diameter (red arrow) was close to the flap diameter (blue arrow). **b** Flap creation in group Flap 2.0: the photodisruption diameters (red arrows) were 0.3 mm larger than the flap diameters (blue arrows)

The VisuMax femtosecond laser system (Carl Zeiss Meditec AG, Jena, Germany) was used to create all LASIK flaps with a pulse repetition rate of 500 kHz and an energy of 130 nJ. The standard of flap diameter was set with 8.1 mm, and the flap thickness was set from 80 to 120 μm. The hinge width was 3.89 mm and the hinge angle was 55° in superior position. The laser shots were created in a spiral-in laser firing pattern with flap track and spot separation at 4.5 µm and the flap side track and spot distance were set to 2 µm. The computer was programmed with the medium cone (M cone) and the surgery was performed with the small cone (S cone). The flaps were created using two different techniques. In group Flap 1.0, the flap diameters were 8.1 mm, and the photodisruption diameters were close to the flap (Fig. 1a). In group Flap 2.0, the interlamellar photodisruption diameters were increased by 0.3 mm, to about 8.4 mm, with 8.1 mm flap diameters (Fig. 1b). Visumax Flap 2.0 program was a modification of the laser programming introduced by the manufacturer Zeiss by expanding the diameter of the interlamellar photodisruption parameters. Table 1 shows laser settings for flap creation in two programs with the VisuMax femtosecond laser. Following flap creation, excimer laser ablation was conducted with a Wavelight EX500 (Wavelight GmbH, Erlangen, Germany).

**Table 1:** The VisuMax femtosecond laser settings for flap creation

Setting	Group Flap 1.0	Group Flap 2.0
Spot distance (μm)	4.5	4.5
Track distance (μm)	4.5	4.5
Pulse energy (nJ)	130	130
Hinge position	Superior	Superior
Scan direction	Spiral in	Spiral in
Scan mode	Single	Single
Flap thickness (μm)	80–120	80–120
Flap diameter (mm)	8.1	8.1
Photodisruption diameters (mm)	8.1	8.4

### Assessment of OBLs

**Fig. 2:**
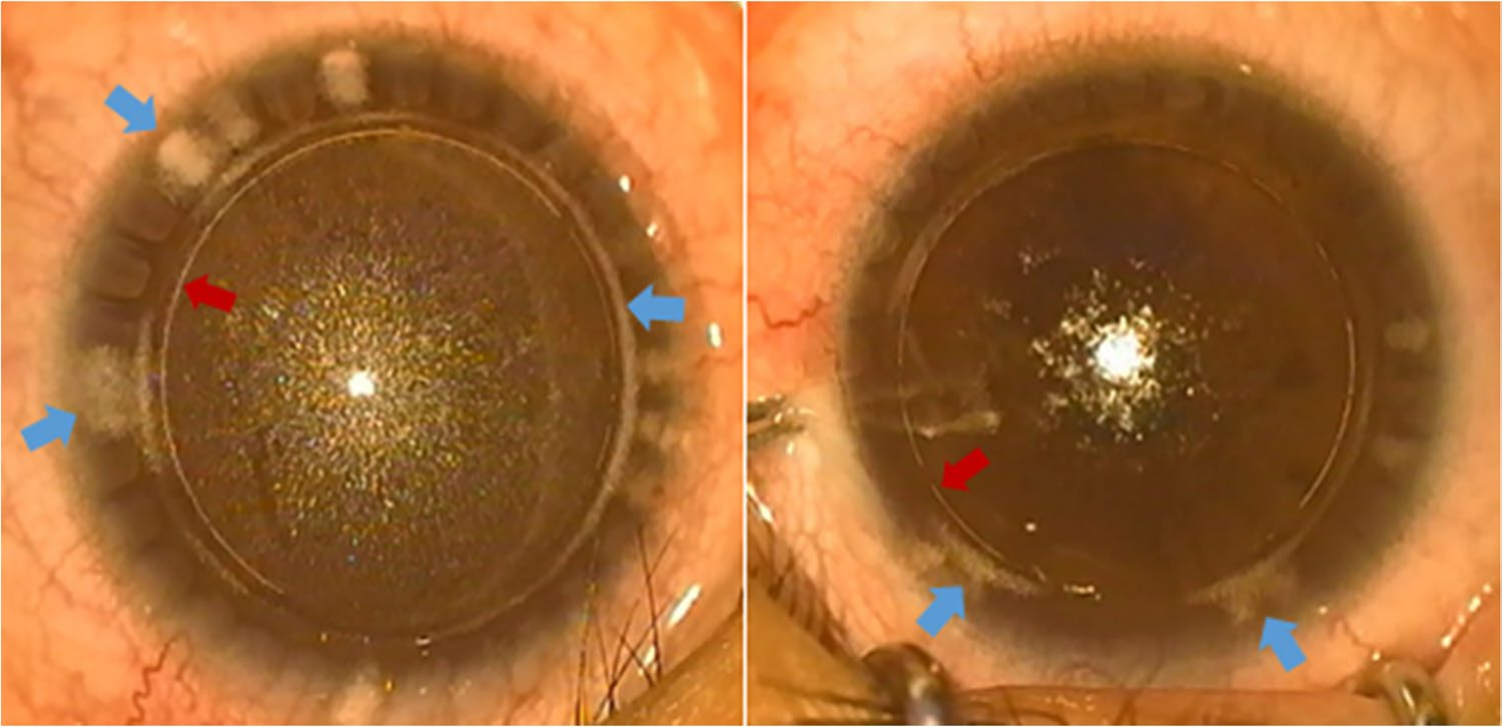
An OBL (blue arrow) outside the corneal flap (red arrow)

All of the surgical procedures were recorded, and screen captures were taken when flap creation was finished. Two investigators independently observed the appearance and types of the OBL in a double-blind situation. When the investigators disagreed, they checked the screen captures together and reached a consensus. Then, they analyzed the area of the OBL by the percentage of pixels where the cornea was indicated in white using ImageJ software (National Institutes of Health). In addition to the OBL inside the corneal flap, the OBL also exists outside the flap, which does not affect the surgical procedure (Fig. 2). Therefore, we only discuss the OBL inside the corneal flap.

### Statistical analysis

All statistical analyses were performed using SPSS software (version 26.0, IBM Corporation). Measurements of visual acuity were converted to log MAR for statistical analysis. The Kolmogorov–Smirnov test was used to confirm the normal distribution of the variables. Continuous variables were described as mean ± standard deviation (SD) using parameter test; otherwise, nonparametric tests were used. Student’s *t-*test was used to compare preoperative parameters between group Flap 1.0 and group Flap 2.0. Statistically significant parameters during the no OBL, hard OBL, and soft OBL groups were considered to be potential risk factors for OBLs in analysis of variance (ANOVA). A multivariate logistic regression analysis was performed to find the associated factors with hard OBLs in the group Flap 2.0 by determining odds ratios (OR) and 95% CI. All statistical significance was set at a *P* value of less than 0.05.

## Results

The study enrolled 1400 eyes of 715 consecutive patients. Of the eyes, 912 (65.1%) were of male patients and 488 (34.9%) were of female patients. Table 2 shows the baseline characteristics of the patients. There were no statistical differences in the preoperative parameters between group Flap 1.0 and group Flap 2.0.

**Table 2:** Preoperative parameters of eyes using the Flap 1.0 and Flap 2.0 program

Parameter	Group Flap 1.0	Group Flap 2.0	*P*
Age (year)	19.99 ± 3.61	20.56 ± 4.46	0.06
Sphere (D)	− 5.09 ± 2.11	− 5.29 ± 2.04	0.06
Cylinder (D)	− 1.36 ± 0.93	− 1.35 ± 0.97	0.64
IOP (mmHg)	15.77 ± 2.54	17.16 ± 2.60	0.40
ISV	20.03 ± 5.93	20.20 ± 6.39	0.13
IVA	0.12 ± 0.05	0.12 ± 0.06	0.08
IHD	0.010 ± 0.006	0.013 ± 0.038	0.14
Flat K (D)	42.05 ± 1.25	42.16 ± 1.36	0.12
Steep K (D)	43.73 ± 1.44	43.86 ± 1.58	0.16
Mean K (D)	42.87 ± 1.28	42.97 ± 1.40	0.10
Axis (°)	89.74 ± 18.59	90.45 ± 14.16	0.36
CCT (mm)	536.97 ± 31.68	533.21 ± 30.47	0.34
Cornea volume (mm^3^)	60.61 ± 3.36	60.49 ± 18.90	0.18
Chamber volume (mm^3^)	205.98 ± 30.49	213.40 ± 35.27	0.48
PD (mm)	3.55 ± 0.73	3.40 ± 0.71	0.43
Flap thickness	90.66 ± 5.98	91.37 ± 8.29	0.06

*IOP*, intraocular pressure; *ISV*, index of surface variance; *IVA*, index of vertical asymmetry; *IHD*, index of height decentration; *K*, keratometry; *CCT*, central corneal thickness; *PD*, pupil diameter

**Fig. 3:**
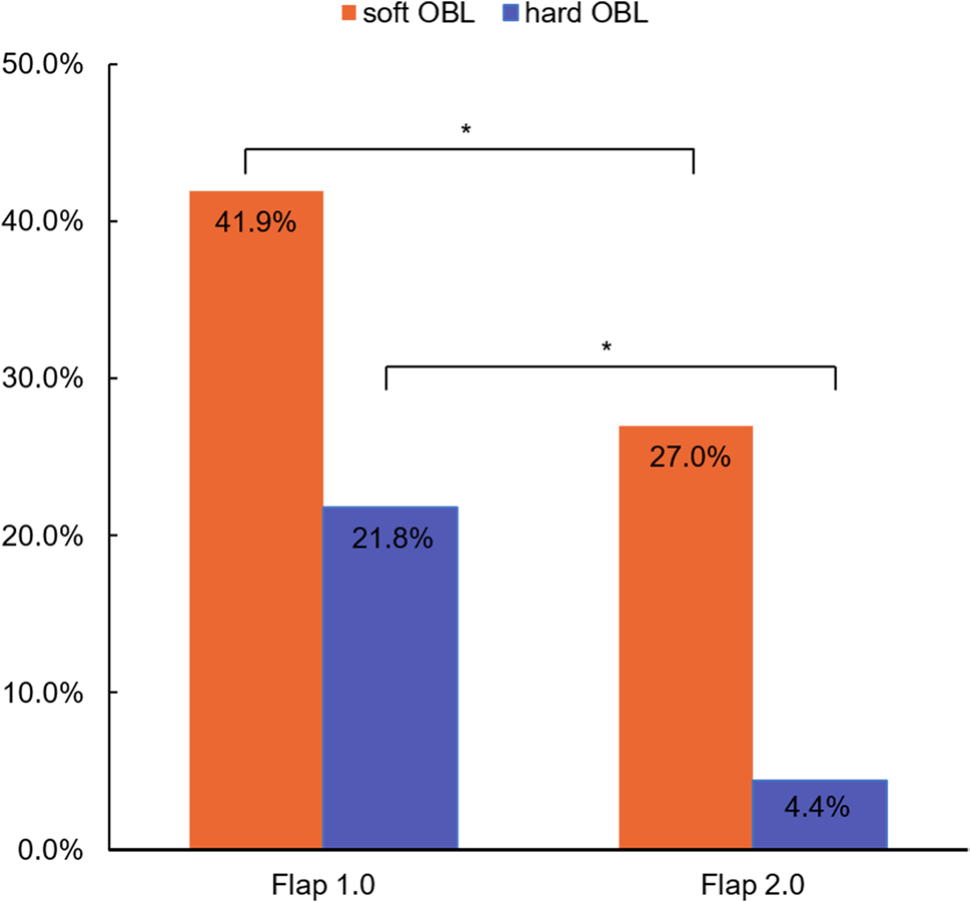
Histograms comparing the percentage of the incidence of a hard and soft OBL within the Flap 1.0 and the Flap 2.0 program. *P < 0.05

Six hundred and seventy-one eyes were treated with the Flap 1.0 program and 729 eyes were treated with the Flap 2.0 program. Four hundred and twenty-seven eyes (63.7%) developed an OBL using the Flap 1.0 program, including 146 eyes that (21.8%) developed a hard OBL and 281 eyes (41.9%) that developed a soft OBL. In group Flap 2.0, 229 eyes (31.4%) developed an OBL, including 32 eyes (4.4%) with a hard OBL and 197 eyes (27.0%) with a soft OBL (Fig. 3).

The hard OBLs created by the Flap 1.0 program covered a mean of 14.62% ± 8.5% of the corneal flap, and those created by the Flap 2.0 program covered a mean of 10.98% ± 6.21% (*P* = 0.007). The soft OBLs created by the Flap 1.0 program covered a mean of 6.62% ± 7.02% of the corneal flap, and those created by the Flap 2.0 program covered a mean of 3.50% ± 3.77% (*P* < 0.001). The incidence and the area of OBLs in both hard OBL and soft OBL groups were significantly lower with the Flap 2.0 program than with the Flap 1.0 program.

In group Flap 2.0, 229 eyes that developed an OBL and 500 eyes in which did not develop an OBL were included. Table 3 compares the preoperative and intraoperative characteristics and postoperative dates in eyes with and without OBLs using the Flap 2.0 program.

**Table 3:** Preoperative and intraoperative characteristics and postoperative dates in eyes without and with hard and soft OBL using the Flap 2.0 program

Characteristic	No OBL group	Hard OBL group	Soft OBL group	*P* value
Preoperative				
Age (year)	20.36 ± 4.48	20.78 ± 4.16	21.04 ± 4.60	0.60
IOP (mmHg)	17.04 ± 2.54	17.44 ± 2.70	17.48 ± 2.82	0.13
Sphere (D)	− 5.05 ± 1.98	− 6.95 ± 1.52	− 5.70 ± 1.90	< 0.001*
Cylinder (D)	− 1.27 ± 0.94	− 1.73 ± 0.98	− 1.49 ± 1.03	0.002*
Flat K (D)	42.01 ± 1.37	43.03 ± 1.15	42.40 ± 1.40	< 0.001*
Steep K (D)	43.64 ± 1.55	44.89 ± 1.49	44.23 ± 1.54	< 0.001*
CCT (mm)	532.89 ± 31.24	535.44 ± 25.55	533.68 ± 29.33	0.87
WTW (mm)	11.84 ± 0.41	11.79 ± 0.35	11.77 ± 0.37	0.13
PD (mm)	3.44 ± 0.72	3.33 ± 0.62	3.37 ± 0.68	0.46
Intraoperative				
Flap thickness (mm)	92.22 ± 8.64	86.09 ± 3.96	90.23 ± 7.53	< 0.001*
Postoperative				
UDVA (logMAR)				
1 day postop	0.03 ± 0.07	0.07 ± 0.08	0.04 ± 0.07	0.002*
10 days postop	0.00 ± 0.06	0.02 ± 0.05	0.01 ± 0.05	0.11
1 month postop	0.00 ± 0.05	0.02 ± 0.07	0.01 ± 0.06	0.17
3 months postop	− 0.02 ± 0.05	0.02 ± 0.07	− 0.01 ± 0.06	0.14
IOP (mmHg)				
1 day postop	13.22 ± 7.76	12.21 ± 3.33	12.23 ± 3.29	0.19
10 days postop	12.16 ± 2.63	11.86 ± 2.97	12.87 ± 5.90	0.10
1 month postop	11.70 ± 2.19	11.59 ± 2.46	12.12 ± 2.53	0.25
3 months postop	11.56 ± 2.10	11.39 ± 2.01	11.97 ± 2.50	0.52

*IOP*, intraocular pressure; *K*, keratometry; *CCT*, central corneal thickness; *WTW*, white-to-white distance; *PD*, pupil diameter; *UDVA*, uncorrected distance visual acuity

*P < 0.05

Using the Flap 2.0 technique, eyes that developed an OBL, especially those with a hard OBL, exhibited the following characteristics that distinguished them from individuals who did not develop an OBL: higher sphere (*P* < 0.001), higher flat keratometry (*P* < 0.001), higher steep keratometry (*P* < 0.001), and thinner flap thickness (*P* < 0.001). Eyes that developed either a hard or soft OBL had more cylinder (*P* = 0.002) (Table 3).

The following parameters were used in a multivariate logistic regression model: sphere, cylinder, flat keratometry, steep keratometry, and flap thickness. Multivariate logistic regression showed that a thinner flap (odds ratio = 1.085, 95% CI = 1.005 to 1.172, *P* = 0.038) and a higher sphere (odds ratio = 1.443, 95% CI = 1.159 to 1.796, *P* = 0.001) were the risk factors affecting the chance of the hard OBL occurring. There was no other parameter that had a significant impact on the model.

## Discussion

This study demonstrates that the Flap 2.0 program, a modification technique for flap production, promotes gas venting by enlarging the interlamellar photodisruption size and therefore reduces the incidence and areas of OBL formation. Moreover, features of the cornea and surgical parameters that lead to the occurrence of OBLs in the course of flap creation procedure were identified.

The OBL formation is assumed to be multifactorial as a result of the femtosecond laser photodisruptive mechanism. Asymmetry of corneal diameter, asymmetric off-center suction, and abnormal intraoperative eye movements can contribute to uneven pressure around the bubbles, leading to the bubbles overflowing in one direction and rapidly forming an OBL outside the flap. These patients were excluded from this study. Only OBLs inside the flap were studied.

There are many machines available for performing a FS-LASIK surgery. The incidence of OBL varies from machine to machine. Courtin et al. [15] reported a 48% incidence of OBL with the WaveLight FS200 laser. When using the IntraLase machine, Liu et al. [3] showed that 21 eyes (52.5%) developed an OBL, 40.0% with a hard OBL and 12.5% with a soft OBL. In another study, Kaiserman et al. [11] found that 84 eyes (56.4%) developed an OBL, hard in 24.2% of eyes and soft in 32.2%. Pietil€a et al. [16] showed that OBL developed in 2% of the eyes when the Ziemer model was applied. In the Visumax model, Lim et al. [17] found that the incidence of OBL was 72.59%. Wu et al. [14] showed that the hard OBL incidence was 28.8% in one group and 7.6% in another group, with both using the Visumax model. In our study of Visumax Flap 2.0 group, 229 eyes (31.4%) developed an OBL, including 32 eyes (4.4%) with a hard OBL and 197 eyes (27.0%) with a soft OBL. The incidence of an OBL in our study appears to be high due to our inclusion of a large number of soft OBLs. Previous studies have revealed that there is no clinical significance to a soft OBL [3, 11], Therefore, focusing only on the incidence of a hard OBL, it was rather low in our study. In addition, the incidence of OBL does not affect the efficacy and safety of the procedure or postoperative visual outcome [3, 11, 16, 17].

Each machine has unique parameters, so there are different ways to reduce the incidence of OBL when using these machines. The WaveLight FS200 femtosecond laser machine creates a canal in the hinge of the flap as a gas-diffusing passage to reduce the incidence of OBLs [18, 19]. A previous study showed that the risk of OBLs reduced significantly due to the wider canal settings along with optimized denser spot application carried out in LASIK flap creation [20]. The IntraLase machine creates a bag that can store gas in order to lower the incidence of OBL [18]. The Ziemer LDV machine uses the low energy pulse, which can reduce bubble formation during the cutting process [21]. In addition, it cuts the flap margin first, eliminating gas bubbles through the incision edge [18]. The Zeiss machine scans from the corneal periphery to the center in the pattern, allowing the gas to eliminate through the margin of the cornea [18]. It uses less energy and requires less suction pressure, so the OBL tends to remain in the corneal stroma [22]. In our study, the incidence of OBLs using Zeiss machine was 31.4% in group Flap 2.0, much lower than the 63.7% in group Flap 1.0, showing the newer program’s clear reduction of OBL production by enlarging the interlamellar photodisruption size.

Mastropasqua et al. [1] studied the flap creation with the same laser design parameters and different presetting diameters of 7.90 mm, 8.0 mm, and 8.20 mm, demonstrating that increasing flap diameter settings, making the flap edge close to the contact glass border, which can reduce the occurrence of OBLs effectively. Liu et al. [3] also demonstrated that increased OBL production was linked to a decreased flap diameter. Wu et al. [14] used the small cone for procedure as well as a medium cone in computer program setting for flap creation, and they found that using this cone modification reduces the formation of OBL significantly.

In our study, we adjusted the intraoperative parameter that used the same flap diameter as before, and enlarged the femtosecond laser interlamellar photodisruption diameters, making the interlamellar photodisruption edge close to the contact glass border. This method (Flap 2.0) is conducive to the discharge of bubbles to the surrounding area, and makes it less likely that bubbles will be accumulated, reducing the incidence of OBLs in general when compared to using the original Flap 1.0 program. This strategy is effective in minimizing OBLs, especially hard OBL formation. The incidence of OBL formation is 31.4%, including hard OBL formation at only 4.4%. This is lower than previously reported percentages in the relevant literature, which ranged from 48 up to 77% [12, 15].

Lim et al. [23] found that in the 80-µm flap group, OBLs were more common than in the 120-µm flap group. In their subsequent study, they found that flap thickness more than 80 µm had a lower incidence of OBLs compared to that of 80 µm [17]. This is consistent with our findings, compared to the no OBL group. The thinner the flap, the more likely it is to produce the hard OBL. Compared the soft OBL group and no OBL group, and the thicker the flap, the less likely it is to develop an OBL.

The lamellar collagen fibers are relatively dense in the corneal anterior stroma adjacent to Bowman’s, making it the compact part of the cornea [24], and as a result, the pressure generated by the negative pressure glass cone makes it more difficult to release gas bubbles. In contrast, the thick corneal flap interface is close to the posterior corneal stroma and the lamina fibers are lax, which is conducive to gas release and dispersion, thus decreasing the chance of OBL formation.

Kaiserman et al. [11] demonstrated that steeper preoperative keratometry values were negatively correlated with the area of OBLs. Jung et al. [22] found that a steep cornea could be a risk factor for an OBL. Courtin et al. [15] showed that there was no difference in flat keratometry and steep keratometry between the OBL and no OBL groups. In our study, high value of steep keratometry and flat keratometry of the anterior corneal surface is more likely to develop OBLs, especially the hard OBL. The reason could be that the cornea is a slightly anteriorly convex flat transverse oval structure, so that the higher the flat keratometry, the more area there is between the cornea and the glass cone in order to suck the cornea. The corneal tissue needs to be compressed by a greater pressure; therefore, hard OBL occurs early on, particularly in the corneal stroma bed close to the cleavage plane. A higher steep keratometry and mean keratometry will both result in the production of either hard or soft OBL, due to the fact that a lower keratometry corneal plane is relatively stressed evenly and less prone to accumulating gas bubbles. More evidence is needed to demonstrate the relationship between different kinds of OBL and corneal curvature parameters.

Previous studies have shown a lower incidence of OBLs in the higher sphere and cylinder group [14, 18]. However, we found that a high sphere is prone to hard OBL. Cylinder is associated with either hard or soft OBLs. The reason could be that a cornea with a large cylinder is difficult to flatten, and with the range of the diameter of photodisruption increased in the Flap 2.0 program, the gas bubbles between the cornea flap and stroma bed appear late and diffuse throughout the cornea, resulting in soft OBL.

In conclusion, the VisuMax Flap 2.0 program, a modification technique for flap creation, decreased the occurrence of OBL formation significantly. An OBL may be more likely to occur if the sphere is higher and the flap thickness is thinner. In addition, the patient’s head position, eye position, and eye rotation during surgery can affect the elimination of gas bubbles, leading to the formation of OBLs. The major limitation of this research is that it is a retrospective study, which is less valid than a study with a randomly assigned control. Further investigations may include personalized intraoperative parameter settings to reduce the risk of an OBL for each individual cornea.
